# Risk Factors for Colorectal Adenoma and Cancer in Comprehensive Health Checkups: Usefulness of Gamma-Glutamyltransferase

**DOI:** 10.3390/jpm14111082

**Published:** 2024-10-30

**Authors:** Yoko Yamanouchi, Maiko Osawa, Takaaki Senbonmatsu, Yuki Shiko, Yohei Kawasaki, Toshihiro Muramatsu

**Affiliations:** 1 Preventive Medicine Center, Saitama Medical University Hospital, Saitama 350-0495, Japan; toshi_m@saitama-med.ac.jp; 2 Department of Rehabilitation Medicine, Saitama Medical University School of Medicine, Saitama 350-0495, Japan; 3 Department of Biostatistics, Graduate School of Medicine, Saitama Medical University, Saitama 350-0495, Japan; m-osawa@saitama-med.ac.jp (M.O.); yshiko@saitama-med.ac.jp (Y.S.); ykawasaki@saitama-med.ac.jp (Y.K.); 4 Research Administration Center, Saitama Medical University, Saitama 350-0495, Japan; senbont@saitama-med.ac.jp

**Keywords:** colorectal adenoma, colorectal cancer, risk factor, gamma-glutamyltransferase, comprehensive health checkup, cancer screening

## Abstract

Background/Objectives: In this study, we aimed to determine the risk factors for colorectal adenoma/cancer by studying patients who underwent comprehensive health checkups and were referred to a hospital because of positive fecal occult blood. Methods: A total of 529 patients were referred to hospital for a positive fecal occult blood test after a comprehensive health checkup at the participating center over a period of 5 years, from January 2018 to December 2022. Patients diagnosed with colorectal adenoma or cancer using colonoscopy were included in the case group, while those diagnosed with no abnormality, diverticulum, or hemorrhoids were included in the control group. Results: Of the 529 referred patients, 503 underwent colonoscopy. A total of 18 colorectal cancers and 191 colorectal adenomas were detected, and there were no tumors, diverticula, or hemorrhoids in any of the 208 patients. Polyps, either hyperplastic or of unknown pathology, were found in 86 patients. A comparison of the case and control groups showed that gamma-glutamyltransferase (GGT) was an independent and significant risk factor for colorectal adenoma or cancer, in addition to previously known risk factors such as male sex, older age, high body mass index, and alcohol consumption. Conclusions: For patients with a positive fecal occult blood test, in addition to traditional risks such as obesity, older age, male sex, and alcohol consumption, identifying those with high GGT levels is recommended to help find colorectal adenoma/cancer.

## 1. Introduction

As dietary habits become more westernized, the incidence of colon cancer has been increasing in the Japanese population [1]. Malignant tumors are the leading cause of death among Japanese adults, and colorectal cancer has the highest incidence and the second highest mortality rate [2,3,4]. Fecal occult blood testing is performed to screen for colorectal cancer. Positive results may suggest cancer, polyps, diverticula, inflammatory bowel disease, and hemorrhoids in the lower digestive tract. Therefore, a positive result indicates the possibility of colon disease, and a detailed examination (colonoscopy) is necessary.

Colorectal cancer arises from colorectal adenomas (adenoma–carcinoma sequence) [5,6,7,8], except in a small percentage of cancers (de novo type). If the disease is treated at the adenoma stage, colorectal cancer can be prevented. Known risk factors for colorectal adenoma/cancer include older age, male sex, height, obesity, lack of exercise, consumption of red and processed meat, alcohol consumption, smoking, and heredity [9,10,11,12,13,14,15]; however, further risk factors should be explored.

Japan employs a special system for the early detection of diseases through comprehensive health checkups. This system is implemented separately from the standardized universal healthcare system and is optionally available for a fee. The primary objectives of a comprehensive health checkup are the early detection of cancer, the detection of metabolic diseases, and the maintenance of health [16,17]. Therefore, several serological tests, including liver and kidney function tests, are performed during comprehensive health checkups. Serum gamma-glutamyltransferase (GGT) is one such serological test performed during a comprehensive health checkup. GGT is widely distributed throughout the body, including the liver, kidneys, pancreas, spleen, and small intestine [18,19,20]. GGT is initially present inside cells, but its value increases when cells are destroyed and it is released into the blood. Even in the absence of a disease, GGT can be detected in the blood, but its value may increase abnormally in the presence of a disease. GGT is generally a marker of liver function and alcohol consumption. However, it has also been linked to metabolic syndrome [21,22,23], oxidative stress, and carcinogenesis [22,24,25,26]. The relationship between colorectal adenomas and GGT levels has recently received increased attention [27,28].

In addition, the association between fatty liver and colorectal adenoma or cancer has not been confirmed, although various theories have been proposed [29,30,31,32,33,34,35,36,37,38,39,40].

Here, we describe a clinical study aimed at evaluating the potential of GGT and fatty liver as diagnostic markers for colorectal adenoma and cancer in patients who underwent colonoscopy and were diagnosed with adenoma or cancer during health checkups.

## 2. Materials and Methods

### 2.1. Ethics Statements

The study design was approved by the Ethics Review Committee of Saitama Medical University Hospital (approval number: 2024-013). As this was a retrospective study, the requirement for informed consent was waived; however, information regarding this study was made public, and the participants were guaranteed the opportunity to refuse participation.

### 2.2. Participants

**Figure 1:**
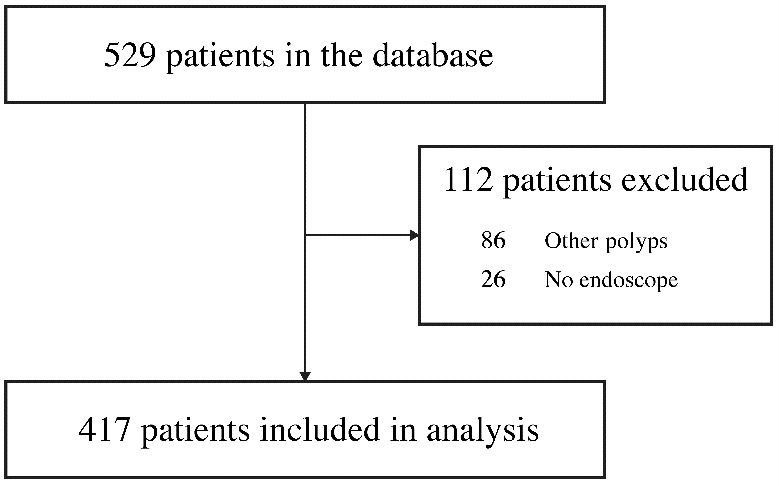
Flow diagram of this study.

A flow diagram of this study is presented in Figure 1. Among the 23,128 examinees who underwent physical examinations at the center over a 5-year period from January 2018 to December 2022, we included 529 patients (mean age: 61.9 years; 335 males and 194 females) aged ≥ 18 years who were referred to various departments of the hospital for fecal occult blood. Patients with coexisting diseases, such as inflammatory bowel disease, were not included in this study.

Patients who underwent a colonoscopy after a hospital referral visit were included in the analysis. Based on the colonoscopy results (including pathology), the subjects were divided into the target group, comprising patients diagnosed with colorectal adenoma (tubular adenoma, tubulovillous adenoma, serrated adenoma) or colorectal cancer (adenocarcinoma, adenocarcinoma in adenoma) using colonoscopy; the control group, including patients without tumors, diverticula, hemorrhoids, or other findings on colonoscopy; and other polyps, including patients with polyps that had not been pathologically examined, hyperplastic polyps, or inflammatory polyps not treated as colorectal adenomas.

### 2.3. Data Collection

For patients who were referred to the hospital with a positive fecal occult blood test during the study period, the following survey items were obtained from the medical records of the center and each hospital department: demographic information, including age and sex; physical findings, including height, weight, body mass index (BMI), and waist circumference; and medical history, including conditions diagnosed at the hospital, conditions under treatment (e.g., diabetes, dyslipidemia, hypertension), smoking habits as indicated by the Brinkman index, drinking habits, and dietary habits (meat, fish, beans, milk products, and vegetables). Laboratory testing included fasting plasma glucose (FPG), hemoglobin A1c, low-density lipoprotein cholesterol, aspartate aminotransferase, alanine aminotransferase, and GGT. Patients fasted for 8 h before blood collection. The collected imaging and physiological findings included the presence of fatty liver on abdominal ultrasound and fecal occult blood test results. Finally, we recorded whether colonoscopy had been performed and, if so, its results, including pathological findings.

### 2.4. Statistical Analyses

Variables are expressed as the mean ± standard deviation. The differences between the two groups were compared using *t*-tests and chi-square tests. Sex, fatty liver, drinking and eating (meat, fish, beans, milk, vegetables) habits, and medical history (treatment for diabetes, dyslipidemia, and hypertension) were considered binary dummy variables. Drinking was classified as “no” for “no drinking” and as “yes” for drinking almost every day, 2–3 times per week, or occasionally. Eating habits were classified as “no” for never eating and as “yes” for occasional or everyday eating for each food type. The Spearman’s correlation coefficient (ρ) was calculated to determine the correlations between factors. Variables were considered to be correlated when the correlation coefficients (ρ) were ≥|0.2|. Multivariate binomial logistic regression analysis was performed, with “patients with adenoma or cancer” and “no adenoma or cancer” as the dependent variables. Independent variables included in the multivariate model were identified as correlated with dependent variables. A stepwise method was used for model selection (*p* < 0.05, included, and *p* > 0.10, removed). Regression coefficients, odds ratios (ORs), 95% confidence intervals (CIs), *p*-values, and variance inflation factors were calculated. Model performance was assessed using the C-index and Hosmer–Lemeshow test. The C-index quantifies how well the model discriminates between positive and negative outcomes [41]. A value of C = 0.5 corresponds to a non-informative prediction rule, whereas C = 1 corresponds to a perfect association [42]. The Hosmer–Lemeshow test was used to assess the model calibration, with small *p*-values indicating that the model is a poor fit. [43]. In addition, GGT levels were converted to binary values using 50 U/L as the cutoff (the reference value of Japan Society of Ningen Dock and Preventive Medical Care) and logistic regression was performed [28]. Subsequently, multivariate binomial logistic regression analysis was performed, with “patients with adenoma” and “patients with cancer” as the dependent variables (adenoma was assigned a status of 0 and cancer was assigned a status of 1). The independent variables were selected and the model was evaluated using the same method. SAS version 9.4 for Windows (SAS Institute, Cary, NC, USA) was used for all statistical analyses.

## 3. Results

### 3.1. Referral Results for Patients with Fecal Occult Blood

During the 5-year period from January 2018 to December 2022, 529 of the 23,128 examinees who underwent comprehensive health checkups were referred to the hospital due to fecal occult blood. After screening for inclusion and exclusion criteria, 112 patients were excluded for the following reasons: other polyps (86 patients) and no endoscopy (26 patients). After screening for exclusions, data from 417 patients were included in the analysis (Figure 1).

Basic information on the 417 included patients is presented in Table 1.

### 3.2. Comparison Between Patients with and Without Adenoma or Cancer

Comparisons between patients with and without adenoma or cancer are presented in Table 2.

The patients with and without adenoma or cancer differed significantly in terms of sex, age, height, weight, BMI, waist circumference, FPG, GGT, fatty liver, alcohol consumption, and Brinkman index. Overall, 156 of the 263 males and 53 of the 154 females had adenoma or cancer (*p* < 0.001). Compared with patients without adenoma or cancer, those with adenoma or cancer were significantly older (64.3 ± 10.6 vs. 59.1 ± 11.9 years, *p* < 0.001), had a greater height (165.0 ± 8.3 vs. 162.1 ± 8.6 cm, *p* < 0.001), greater weight (64.2 ± 11.7 vs. 59.0 ± 10.3 kg, *p* < 0.001), higher BMI (23.7 ± 3.3 vs. 22.4 ± 2.9 kg/m^2^, *p* < 0.001), and smaller waist circumference (78.6 ± 19.6 vs. 79.0 ± 11.6 cm, *p* < 0.001). Regarding laboratory findings, patients with adenoma or cancer had higher FPG (107.1 ± 21.4 vs. 101.7 ± 18.2, *p* = 0.006) and GGT (42.6 ± 43.6 vs. 29.4 ± 37.4 U/L, *p* = 0.001) levels. Additionally, the presence of adenoma or cancer was more common in patients with fatty liver than in those without (81/133 vs. 128/284, *p* = 0.003) and more common in drinkers than in non-drinkers (130/236 vs. 79/181, *p* = 0.021). Finally, the Brinkman index was significantly higher in patients with adenoma or cancer than in those without (229.4 ± 321.3 vs. 152.6 ± 275.4, *p* = 0.009).

### 3.3. Correlation with the Presence of Adenoma or Cancer

The correlations with the presence of adenoma or cancer are presented in Table 3. Sex, age, weight, and GGT levels were weakly correlated with the presence of adenoma or cancer.

### 3.4. Associations with the Presence of Adenoma or Cancer

Sex, age, and GGT levels were correlated with the presence of adenoma or cancer ≥0.2, whereas height (ρ = 0.18), weight (ρ = 0.24), and BMI (ρ = 0.19) had weak correlations. We included BMI in this model because it contains information about height and weight. Accordingly, multivariate binomial logistic regression analysis was performed to determine whether there was an association between sex, age, BMI, and GGT levels and the presence of adenoma or cancer. The association between these variables, and the presence and absence of cancer is presented in Table 4. All variables were selected using the stepwise method. The model demonstrated good discrimination and goodness-of-fit (C-index = 0.717; Hosmer–Lemeshow test, *p* = 0.942). Age (OR = 1.044, *p* < 0.001), sex (OR = 0.494, *p* = 0.002), BMI (OR = 1.127, *p* < 0.001), and GGT levels (OR = 1.008, *p* = 0.016) were significantly associated with the presence of adenoma or cancer.

### 3.5. Association with the Presence of Adenoma or Cancer Using Categorized GGT Values

GGT was considered a binary variable with a cutoff value of 50 U/L. Multivariate binomial logistic regression analysis was performed with the variables included in the previous model (sex, age, and BMI) to categorize GGT levels. The association between these variables and the presence of adenoma or cancer is presented in Table 5. The model showed good discrimination and goodness-of-fit (C-index = 0.715; Hosmer–Lemeshow test, *p* = 0.8552). Age (OR = 1.043, *p* < 0.001), sex (OR = 0.499, *p* = 0.002), BMI (OR = 1.130, *p* = 0.001), and GGT level (OR = 2.470, *p* = 0.006) were associated with the presence of adenoma or cancer.

### 3.6. Comparisons Between Patients with Adenoma and Cancer

Comparisons between patients with adenoma and those with cancer are presented in Table 6. The BMI of patients with cancer was significantly higher than that of patients with adenoma (23.5 ± 3.2 vs. 25.9 ± 3.5 kg/m^2^) (*p* = 0.003), whereas GGT levels were not significantly different between patients with adenoma and those with carcinoma.

### 3.7. Correlations Between the Measured Variables and the Presence of Adenoma and Cancer

The correlations between the measured variables and the presence of adenoma and cancer are presented in Table 7. BMI was weakly correlated with the presence of adenoma and cancer.

### 3.8. Association Between Presence of Adenoma and Cancer

Multivariate binomial logistic regression analysis was performed to determine whether there was an association between BMI and the presence of adenoma and of cancer; the findings are shown in Table 8. We found that BMI (OR = 1.209, *p* = 0.005) was significantly associated with the presence of adenoma and cancer.

## 4. Discussion

In this study, we conducted a risk factor prediction study for colorectal adenoma or cancer during a comprehensive health checkup. Considering that the sample comprised patients with positive fecal occult blood test results, these results are particularly useful in determining high-risk patients for colonoscopy.

Male sex, older age, higher BMI, and alcohol consumption are traditional risk factors for colorectal adenoma and cancer; however, GGT has also been shown to be a risk factor. As mentioned earlier, GGT is not only a marker of liver function and alcohol consumption but has also been linked to metabolic syndrome, oxidative stress, and carcinogenesis [21,22,23,24,25,26]. The relationship between colorectal adenomas and GGT levels has recently received increased attention [27,28]. Elevated GGT levels are naturally assumed to be related to fatty liver disease.

The alcoholic and non-alcoholic causes of fatty liver have been studied; however, there is an overlap between both causes, and it is sometimes difficult to clearly distinguish between the two [44]. Indeed, in this study, we found no distinction between the two.

The association between fatty liver and colorectal adenoma or cancer has been the subject of various studies, but no molecular biology consensus has been reached [29,30,31,32,33,34,35,36,37,38,39,40]. Although previous studies have suggested a link between fatty liver and colorectal adenoma or cancer, the relationship is complex, and other factors may also have an influence. For example, both fatty liver and colorectal adenomas are associated with lifestyle-related diseases, such as obesity and diabetes, which may impact risk.

In this study, fatty liver was significantly more common in the colorectal adenoma or cancer group, but it was not an independent risk factor in the multivariate analysis. Interestingly, GGT levels were shown to be an independent risk factor. In addition to traditional risk factors, GGT should be considered when considering the risk of colorectal adenoma or cancer. When we performed a sensitivity analysis of the relationship between the presence or absence of adenoma or cancer using a cutoff value of 50 U/L as the upper normal limit of GGT, we found a high OR along with previously identified risk factors, suggesting that an elevated GGT level is also an important risk factor for colorectal adenoma or cancer. We also should consider the possibility of confounding the analysis with other diseases that may show elevated GGT levels. At present, it remains unclear whether high GGT levels increase the risk of colorectal adenoma or cancer or whether these conditions induce high GGT levels. Our results are also considered to be influenced by these factors. Therefore, as a next step, we investigated whether high GGT levels (>50 U/L) elicited different colorectal adenoma and cancer risks; however, only BMI was shown to be an independent risk factor. Hong et al. reported that colonic gland cells secrete GGT when transitioning to adenomas [27]. However, there are no reports on changes in serum values during the transition from adenoma to cancer. If these results are correct, then the present data may not be contradictory.

In this study, nearly half of the patients with positive fecal occult blood test results during the comprehensive health checkup had colorectal adenomas. Early detection of cancer is an important objective of comprehensive health checkups [16,17]. Colorectal cancer arises from colorectal adenomas (adenoma–carcinoma sequences) [5,6,7,8], with the exception of a small percentage of cancers (de novo type), and adenomas should be treated at the earliest possible stage before they develop into colorectal cancer. Patients with high GGT levels, in addition to traditional risk factors, such as obesity, older age, male sex, and alcohol consumption, should be encouraged to undergo medical checkups to reduce the incidence of colorectal cancers in the future.

### Study Limitations

First, the participants were exclusively Japanese adults of Asian ethnicity. Second, the recipients of the comprehensive health checkup were considered a group within the Japanese population that is somewhat health-conscious and not economically deprived. Third, fatty liver was diagnosed by abdominal ultrasound and not by a definitive diagnostic method such as liver biopsy. Fourth, drinking was investigated only in terms of the frequency of drinking, not the amount of alcohol consumed. Fifth, dietary habits were also investigated, but only the frequency of food intake and not the quantity thereof. Finally, as both fatty liver and colorectal adenoma or cancer are associated with common factors, such as obesity and diabetes, it was difficult to make independent statistical judgments.

In addition, we did not examine family history for colorectal cancer and/or adenoma and for genetic syndromes (FAP, Lynch Syndrome, etc.) during the interview at the center.

## 5. Conclusions

For patients with fecal occult blood, in addition to traditional risk factors such as obesity, older age, male sex, and alcohol consumption, identifying those with high GGT levels is recommended to help find colorectal adenoma/cancer.

## Figures and Tables

**Table 1:** Basic information on the 417 patients referred to the hospital due to positive fecal occult blood testing.

	Male (*n* = 263)	Female (*n* = 154)
Target group	156	53
Adenoma	142	49
Cancer	14	4
Control group	107	101

Types of adenomas: tubular adenoma, 170; tubulovillous adenoma, 9; tubular adenoma + tubulovillous adenoma, 9; serrated adenoma, 1; serrated adenoma + tubular adenoma, 2.

**Table 2:** Comparison between patients with and without adenoma or cancer.

	Adenoma or Cancer (*n* = 209)	No Adenoma or Cancer (*n* = 208)	*p*-Value
Sex (male/female)	156/53	107/101	<0.001 ^2^
Age (years)	64.3 ± 10.6	59.1 ± 11.9	<0.001 ^1^
Height (cm)	165.0 ± 8.3	162.1 ± 8.6	<0.001 ^1^
Weight (kg)	64.2 ± 11.7	59.0 ± 10.3	<0.001 ^1^
BMI (kg/m^2^)	23.7 ± 3.3	22.4 ± 2.9	<0.001 ^1^
Girth (cm)	78.6 ± 19.6	79.0 ± 11.6	<0.001 ^1^
FPG (mg/dL)	107.1 ± 21.4	101.7 ± 18.2	0.006 ^1^
HbA1c (%)	5.7 ± 0.6	5.6 ± 0.6	0.056 ^1^
LDL-C (mg/dL)	123.1 ± 31.0	123.7 ± 27.0	0.817 ^1^
AST (U/L)	24.5 ± 8.7	24.5 ± 21.3	0.999 ^1^
ALT (U/L)	23.5 ± 13.3	23.3 ± 42.1	0.929 ^1^
GGT (U/L)	42.6 ± 43.6	29.4 ± 37.4	0.001 ^1^
Fatty liver (none/positive)	128/81	156/52	0.003 ^2^
Drinking (no/yes)	79/130	102/106	0.021 ^2^
Brinkman index (number*year)	229.4 ± 321.3	152.6 ± 275.4	0.009 ^1^
Meat (no/yes)	2/207	1/207	0.565 ^2^
Fish (no/yes)	4/205	1/207	0.179 ^2^
Beans (no/yes)	9/200	3/205	0.080 ^2^
Milk (no/yes)	15/194	10/198	0.308 ^2^
Vegetables (no/yes)	1/208	1/207	0.997 ^2^
DM treatment (no/yes)	186/23	194/14	0.125 ^2^
HL treatment (no/yes)	171/38	167/41	0.690 ^2^
HT treatment (no/yes)	142/67	153/55	0.208 ^2^

^1^ *t*-test; ^2^ chi-squared test. BMI: body mass index; FPG: fasting plasma glucose; HbA1c: hemoglobin A1c; LDL-C: low-density lipoprotein cholesterol; AST: aspartate aminotransferase; ALT: alanine aminotransferase; GGT: gamma-glutamyl transferase; HT: hypertension; HL: hyperlipidemia; DM: diabetes mellitus.

**Table 3:** Correlations between measured variables and presence of adenoma/cancer.

	ρ (r)	*p*
Sex (male/female)	−0.24	<0.001
Age (years)	0.22	<0.001
Height (cm)	0.18	<0.001
Weight (kg)	0.24	<0.001
BMI (kg/m^2^)	0.19	<0.001
Waist circumference (cm)	0.17	0.001
FPG (mg/dL)	0.19	<0.001
HbA1c (%)	0.15	0.003
LDL-C (mg/dL)	−0.01	0.790
AST (U/L)	0.11	0.026
ALT (U/L)	0.12	0.011
GGT (U/L)	0.27	<0.001
Fatty liver (none/positive)	0.15	0.003
Drinking (no/yes)	0.11	0.021
Brinkman index (number*year)	0.14	0.005
Meat (no/yes)	−0.03	0.566
Fish (no/yes)	−0.07	0.180
Beans (no/yes)	−0.09	0.081
Milk (no/yes)	−0.05	0.309
Vegetables (no/yes)	0.00	0.997
DM treatment (no/yes)	0.08	0.126
HL treatment (no/yes)	−0.02	0.691
HT treatment (no/yes)	0.06	0.209

BMI: body mass index; FPG: fasting plasma glucose; HbA1c: hemoglobin A 1c; LDL-C: low-density lipoprotein cholesterol; AST: aspartate aminotransferase; ALT: alanine aminotransferase; GGT: gamma-glutamyl transferase; HT: hypertension; HL: hyperlipidemia; DM: diabetes mellitus.

**Table 4:** Logistic regression findings showing the relationship between each variable and the presence or absence of cancer.

	β	OR	95% CI		*p*	VIF
			Lower	Upper		
Age	0.043	1.044	1.024	1.063	<0.001	1.027
Sex (reference: male)	−0.353	0.494	0.317	0.771	0.002	1.094
BMI	0.120	1.127	1.050	1.210	<0.001	1.067
GGT	0.008	1.008	1.001	1.014	0.016	1.037

CI: confidence interval; OR: odds ratio; VIF: variance inflation factor; BMI: body mass index; GGT: gamma-glutamyl transferase.

**Table 5:** Logistic regression findings showing association with the presence of adenoma or cancer using categorized GGT levels.

	β	OR	95% CI		*p*	VIF
			Lower	Upper		
Age	0.043	1.043	1.024	1.063	<0.001	1.025
Sex (reference: male)	−0.347	0.499	0.321	0.778	0.002	1.100
BMI	0.123	1.130	1.053	1.213	0.001	1.063
GGT (reference: ≤50)	0.452	2.470	1.301	4.688	0.006	1.037

CI: confidence interval; OR: odds ratio; VIF: variance inflation factor; BMI: body mass index; GGT: gamma-glutamyl transferase.

**Table 6:** Comparison between patients with adenoma and cancer.

	Adenoma (*n* = 191)	Cancer (*n* = 18)	*p*-Value
Sex (male/female)	142/49	14/4	0.749 ^2^
Age (years)	64.4 ± 10.8	62.7 ± 7.7	0.519 ^1^
Height (cm)	165.2 ± 7.9	162.9 ± 11.5	0.253 ^1^
Weight (kg)	63.8 ± 11.6	68.9 ± 12.0	0.078 ^1^
BMI (kg/m^2^)	23.5 ± 3.2	25.9 ± 3.5	0.003 ^1^
Waist circumference (cm)	79.1 ± 17.9	73.3 ± 32.4	0.231 ^1^
FPG (mg/dL)	106.0 ± 16.3	118.3 ± 50.0	0.020 ^1^
HbA1c (%)	5.7 ± 0.6	5.8 ± 0.8	0.463 ^1^
LDL-C (mg/dL)	123.5 ± 30.9	118.5 ± 32.3	0.514 ^1^
AST (U/L)	24.4 ± 8.3	26.2 ± 11.6	0.390 ^1^
ALT (U/L)	23.2 ± 13.4	26.7 ± 11.8	0.289 ^1^
GGT (U/L)	43.2 ± 45.2	35.7 ± 19.4	0.483 ^1^
Fatty liver (none/positive)	120/71	8/10	0.126 ^2^
Drinking (no/yes)	71/120	8/10	0.543 ^2^
Brinkman index (number*year)	222.8 ± 318.6	298.8 ± 350.9	0.339 ^1^
Meat (no/yes)	2/189	0/18	0.663 ^2^
Fish (no/yes)	3/188	1/17	0.238 ^2^
Beans (no/yes)	8/183	1/17	0.785 ^2^
Milk (no/yes)	15/176	0/18	0.217 ^2^
Vegetables (no/yes)	1/190	0/18	0.758 ^2^
DM treatment (no/yes)	171/20	15/3	0.422 ^2^
HL treatment (no/yes)	159/32	12/6	0.081 ^2^
HT treatment (no/yes)	133/58	9/9	0.088 ^2^

^1^ *t*-test; ^2^ chi-squared test. BMI: body mass index; FPG: fasting plasma glucose; HbA1c: hemoglobin A1c; LDL-C: low-density lipoprotein cholesterol; AST: aspartate aminotransferase; ALT: alanine aminotransferase; GGT: gamma-glutamyl transferase; HT: hypertension; HL: hyperlipidemia; DM: diabetes mellitus.

**Table 7:** Correlation with the presence of adenoma and of cancer.

	ρ (r)	*p*
Sex (male/female)	−0.02	0.750
Age (years)	−0.07	0.308
Height (cm)	−0.07	0.296
Weight (kg)	0.14	0.038
BMI (kg/m^2^)	0.21	0.003
Waist circumference (cm)	0.09	0.219
FPG (mg/dl)	0.04	0.596
HbA1c (%)	0.03	0.703
LDL-C (mg/dl)	−0.06	0.393
AST (U/L)	0.04	0.524
ALT (U/L)	0.13	0.057
GGT (U/L)	0.04	0.579
Fatty liver (none/positive)	0.11	0.127
Drinking (no/yes)	−0.04	0.545
Brinkman index (number*year)	0.06	0.380
Meat (no/yes)	0.03	0.665
Fish (no/yes)	−0.08	0.240
Beans (no/yes)	−0.02	0.786
Milk (no/yes)	0.09	0.219
Vegetables (no/yes)	0.02	0.760
DM treatment (no/yes)	0.06	0.424
HL treatment (no/yes)	0.12	0.082
HT treatment (no/yes)	0.12	0.09

BMI: body mass index; FPG: fasting plasma glucose; HbA1c: hemoglobin A1c; LDL-C: low-density lipoprotein cholesterol; AST: aspartate aminotransferase; ALT: alanine aminotransferase; GGT: gamma-glutamyl transferase; HT: hypertension; HL: hyperlipidemia; DM: diabetes mellitus.

**Table 8:** Association between BMI and the presence of adenoma and of cancer.

	β	OR	95% CI		*p*
			Lower	Upper	
BMI	0.191	1.209	1.060	1.380	0.005

CI: confidence interval; OR: odds ratio; BMI: body mass index.

## Data Availability

The original contributions presented in this study are included in the article. Further inquiries can be directed to the corresponding author.
